# Psychiatric symptoms in people living with HIV: prevalences, interactions and consequences

**DOI:** 10.1192/j.eurpsy.2023.1102

**Published:** 2023-07-19

**Authors:** E. M. Meeder, M. Blaauw, L. E. van Eekeren, A. Groenendijk, W. A. Vos, Q. de Mast, W. L. Blok, A. Verbon, M. A. Berrevoets, J. van Lunzen, L. Joosten, M. Netea, V. Matzaraki, A. J. van der Ven, A. F. Schellekens

**Affiliations:** 1 Psychiatry; 2 Internal Medicine, Radboudumc, Nijmegen; 3 Internal Medicine, OLVG, Amsterdam; 4 Internal Medicine, Erasmus MC, Rotterdam; 5 Internal Medicine, Elisabeth-Tweesteden Ziekenhuis, Tilburg, Netherlands; 6 ViiV Healthcare, London, United Kingdom

## Abstract

**Introduction:**

People living with HIV (PLHIV) experience higher levels of mental health issues compared to the general population. Especially depression, anxiety, impulsivity and substance use occur frequently in PLHIV. This is thought to have important consequences for quality of life, sexual risk behaviour and antiretroviral treatment (ART) adherence. Both in PLHIV as well as in the general population, divergent psychiatric symptoms often co-occur, and influence one another.

**Objectives:**

To assess the interrelatedness of psychiatric symptoms and their potential consequences in PLHIV.

**Methods:**

Data from 1615 outpatient PLHIV using suppressive ART from the 2000HIV study (NCT03994835) were analysed. Participants reported on the severity of substance use (MATE-Q), depression and anxiety (HADS), impulsivity (BIS-11), quality of life (EQ-5D-5L), ART adherence (MASS-8) and sexual risk behaviour. For these variables, prevalence rates and mean scores were calculated. After binarizing the data, an Ising network model was constructed. Using this network, interrelations between psychiatric symptoms were assessed, the centrality of symptoms was estimated and connections with clinical consequences were explored.

**Results:**

In our cohort of PLHIV, the increased prevalence of substance use was most pronounced, as shown by a prevalence rate of 28.7% for smoking, 13.6% for cannabis use, 11.1% for heavy alcohol drinking and 9.2% for ecstasy use in the past month. The network analysis revealed that symptoms of depression and anxiety were most strongly interrelated. The depressive symptom “feeling slowed down” was one of the most central symptoms, and was most strongly connected with quality of life. Substance use was associated with a higher occurrence of sexually transmitted diseases, and this relationship was mediated by a higher number of sexual partners. Notably, ART adherence did not display any connections with depression, anxiety, impulsivity or substance use.

**Image:**

**Figure:**
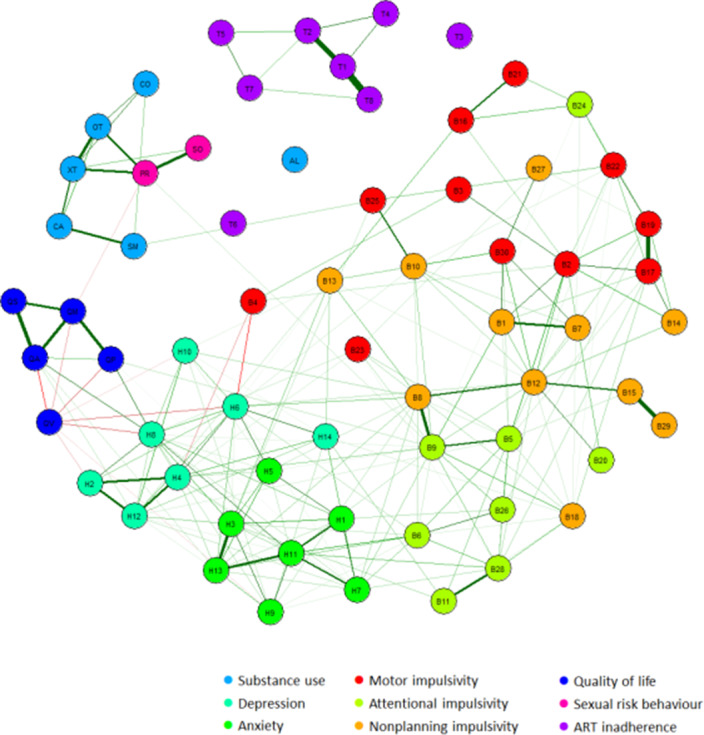

**Conclusions:**

The high occurrence of substance use and its link with sexual risk behaviour, emphasizes it’s role as a potential target for prevention of HIV transmission. Contrary to general assumption, psychiatric symptoms are not associated with lower levels of ART adherence in our cohort. Treatment of depression in PLHIV might be improved by focussing on the symptom of feeling slowed down, since this symptom was most strongly connected with quality of life.

**Disclosure of Interest:**

E. Meeder Grant / Research support from: ViiV Healthcare, M. Blaauw: None Declared, L. van Eekeren: None Declared, A. Groenendijk: None Declared, W. Vos: None Declared, Q. de Mast: None Declared, W. Blok: None Declared, A. Verbon: None Declared, M. Berrevoets: None Declared, J. van Lunzen Employee of: ViiV Healthcare, L. Joosten: None Declared, M. Netea: None Declared, V. Matzaraki: None Declared, A. van der Ven: None Declared, A. Schellekens: None Declared

